# Comparison between open and closed reduction techniques for mandibular fractures: A systematic review

**DOI:** 10.6026/973206300213451

**Published:** 2025-10-31

**Authors:** Krishna Prasad Biswas, Divashree Sharma, Monica Chaurasia, Neelam Shakya, Vidhya Balke, Alisha Alam, Rashmi Laddha

**Affiliations:** 1 Department of Dentistry (Conservative Dentistry and Endodontics), AIIMS, Guwahati, Assam, India; 2 Department of Dentistry, Government Medical College, Satna, Madhya Pradesh, India; 3 Department of Dentistry, Government Medical College, Satna, Madhya Pradesh, India; 4 Department of Oral and Maxillofacial Surgery, Government Dental College, Indore, Madhya Pradesh, India; 5 Department of Oral and Maxillofacial Surgery, Government Dental College, Indore, Madhya Pradesh, India; 6 Department of Oral and Maxillofacial Surgery, Jaipur Dental College, Rajasthan, India

**Keywords:** Mandibular fractures, condylar fractures, open reduction, closed reduction, Internal Fixation, functional outcomes

## Abstract

The ideal management of mandibular fractures is still controversial and both closed reduction and open reduction procedures are practiced extensively. The objective of this systematic review was to compare and assess the functional outcome, complication rate, esthetic outcome and patient satisfaction in both forms of treatment. Exhaustive searching of various databases was done to select studies that compared open with closed reduction directly for treating mandibular fractures. The evidence supports that open reduction is superior in early functional recovery, reduced immobilization and improved aesthetics, while closed reduction has advantages of more prolonged treatment but less severe surgical complications. Both are nonetheless effective in gaining successful fracture healing if properly chosen according to the clinical condition of patients.

## Background:

Mandibular fractures represent a frequent percentage of maxillofacial traumas and are caused by high-energy trauma in the guise of road traffic crashes, interpersonal violence, trauma related to sports and falls [1]. Mandible's exposed anatomical location and solitary structural characteristics make it prone to fracture in different areas such as symphysis, parasymphysis, body, angle, ramus and condylar processes [2]. As the mandible is both esthetically and functionally important, early and effective treatment of such fractures is crucial to restore occlusion, mastication, facial balance and avoid morbidities of long-standing nature [3]. Fractures of mandible account for a considerable proportion of maxillofacial fractures, primarily resulting from high-energy trauma including road traffic accidents, interpersonal violence, injuries due to sports and falls [4]. Mandible's well-situated anatomical position and special structural features render it susceptible to fractures in different areas like symphysis, parasymphysis, body, angle, ramus and condylar processes [5]. As the mandible is of functional and esthetic significance, early and adequate management of such fractures is essential to re-establish occlusion, mastication, facial symmetry and avoid long-term morbidities [6]. Conversely, open reduction and internal fixation (ORIF) has become increasingly popular with improvements in surgical technique, rigid fixation hardware and anesthesia protocol [7]. ORIF enables direct visual control over the area of fracture, accurate anatomical reduction and point fixation using miniplates and screws [8]. It enables early functional rehabilitation, reduced immobilization time and enhanced patient comfort. Open reduction has all these benefits despite them. Open reduction has some risks of surgery, such as hardware issues, infection and scarring [9]. Whether to use open or closed reduction methods is usually decided based on many factors like pattern and location of fracture, grade of displacement, skill of the surgeon, available facilities and patient-related factors [10]. Even though extensive research has been done comparing the outcome of these treatment modalities, heterogeneity regarding study design, patient groups and outcome measures has led to different conclusions [11]. The present systematic review attempts to synthesize the existing evidence between open and closed reduction techniques in treating mandibular fractures [12]. Therefore, it is of interest to evaluate of functional outcomes, complication rates, aesthetic results, immobilization duration, patient satisfaction and overall clinical efficiency in an attempt to have a comprehensive understanding that can potentially help guide clinical decision-making.

## Materials and Methods:

Systematic research was conducted to search for research studies comparing the closed reduction techniques with open reduction and internal fixation of treatment of mandibular fractures. Electronic databases used included PubMed, Scopus, Web of Science and Google Scholar, with publication dates from January 2000 to December 2023. Keywords and medical subject headings (MeSH) for the search were "mandibular fractures," "open reduction," "closed reduction," "internal fixation," "maxillomandibular fixation," "functional outcome," and "complications." Studies were included if they explicitly compared the two treatment modalities and communicated clinical outcomes like fracture healing, functional recovery, complications, aesthetic outcomes, or patient satisfaction. Randomized controlled trials, prospective and retrospective comparative studies and well-documented observational studies were included. Case reports, technical notes, editorials, conference abstracts and studies without comparative data were excluded. Non-English articles were also excluded. Titles and abstracts of potential articles were independently screened and full texts of potentially relevant studies were obtained for careful examination. Information was retrieved on study design, sample size, patients' demographics, types of fractures, treatment strategy and outcomes. As a result of heterogeneity in study designs and outcome, qualitative analysis was conducted and descriptive synthesis was presented to provide an overview of the evidence available.

## Results:

A total of 22 studies were included in this systematic review after screening the available literature based on the inclusion and exclusion criteria. The selected studies encompassed a variety of study designs, including randomized controlled trials, prospective cohort studies and retrospective comparative analyses, involving patients managed with either open reduction and internal fixation or closed reduction techniques for mandibular fractures. The total number of patients evaluated across these studies exceeded 1200, with age groups ranging predominantly between 18 and 60 years and a clear male predominance consistently observed. The most common etiological factor for mandibular fractures was road traffic accidents, followed by assaults and falls. Across the included studies, parasymphysis, body, angle and condylar regions were reported as the most frequent anatomical sites of fracture. The review findings demonstrated that open reduction provided advantages in terms of early functional recovery, quicker return to normal diet, better aesthetic outcomes and shorter duration of immobilization and hospitalization. In contrast, closed reduction was associated with prolonged immobilization but offered the advantage of avoiding surgical exposure, thereby reducing operative risks. Complication rates, including infection, malocclusion and hardware-related issues, were variable but generally low across both groups when appropriate protocols were followed.

Table 1 (see PDF) shows the distribution of studies included based on their study design. Table 2 (see PDF) shows the summary of the reported age distribution of patients in the included studies. Table 3 (see PDF) shows the gender distribution reported in the included studies. Table 4 (see PDF) shows the most common causes of mandibular fractures reported in the literature. Table 5 (see PDF) shows the most commonly involved fracture sites as reported across studies. Table 6 (see PDF) shows the average operative time reported for both techniques. Table 7 (see PDF) shows the mean hospital stay for each technique. Table 8 (see PDF) shows the summary of the average immobilization periods reported. Table 9 (see PDF) shows the proportion of patients achieving early functional recovery. Table 10 (see PDF) shows the summary of the aesthetic outcomes based on reported patient and surgeon assessments. Table 11 (see PDF) shows the outline of the reported complication rates across both techniques. Table 12 (see PDF) shows the patient satisfaction levels reported in the studies. Table 1 (see PDF) shows the distribution of studies based on study design. Table 2 (see PDF) and Table 3 (see PDF) summarize the age and gender distribution across studies. Table 4 (see PDF) identifies road traffic accidents as the leading etiology. Table 5 (see PDF) highlights parasymphysis as the most frequent fracture site. Table 6 (see PDF) and Table 7 (see PDF) show shorter operative time and hospital stay for open reduction. Table 8 (see PDF) reflects longer immobilization periods with closed reduction. Table 9 (see PDF) shows superior functional recovery in open reduction. Table 10 (see PDF) demonstrates better aesthetic outcomes with open reduction. Table 11 (see PDF) presents comparable but slightly higher complication rates in closed reduction. Table 12 (see PDF) shows higher patient satisfaction in the open reduction group.

## Discussion:

Mandibular fractures remain among the most frequently encountered injuries in maxillofacial trauma, often requiring careful evaluation and individualized management to optimize both functional and aesthetic outcomes [13]. The choice between open and closed reduction techniques continues to generate clinical debate, as both approaches offer distinct advantages and limitations depending on multiple factors such as fracture location, displacement, patient condition and available resources [14]. The present systematic review comprehensively evaluated the available evidence comparing open reduction and internal fixation with closed reduction methods for the treatment of mandibular fractures [15]. The majority of studies consistently reported that open reduction allows for direct visualization of fracture segments, accurate anatomical realignment and rigid internal fixation, leading to early mobilization, faster restoration of masticatory function and quicker return to daily activities [16]. These advantages directly contribute to improved patient comfort and higher satisfaction levels. Furthermore, open reduction minimizes the need for prolonged maxillomandibular fixation, which is often considered inconvenient by patients, particularly in terms of nutritional restrictions, speech difficulties, oral hygiene challenges and overall quality of life during recovery [17]. Aesthetic results were also said to be better with open reduction. Accurate anatomical reduction allows for improved facial symmetry and alignment, reducing postoperative deformity or contour irregularities that can result from closed reduction not being able to place fractures in optimal position [18]. In addition, advances in surgical technique and the increasing use of intraoral approaches in most open reduction procedures have reduced visible scarring, further increasing aesthetic satisfaction [19]. Yet, open reduction is not free from its adverse implications. Surgical procedures involve risks inherent in them, such as infection, nerve damage, exposure of the hardware and intraoperative complications [20]. However, most studies in this review showed that if adequate surgical techniques and aseptic measures are adhered to, rates of complications related to open reduction are low and controllable [21]. Closed reduction, by contrast, still has a valid place in properly selected fracture patterns, especially for non-displaced or minimally displaced fractures in which surgical exposure becomes unnecessary. The fact that closed reduction is not an invasive procedure minimizes operative morbidity and obviates hardware-related complications [22]. Nevertheless, the need for prolonged immobilization usually translates into longer treatment time, functional recovery retardation and patient inconvenience [23]. In some instances, also closed reduction can be related to residual malocclusion or poor anatomical alignment if fracture fragments are not stabilized in the best possible manner [24]. Overall, the evidence from this systematic review shows that both open and closed reduction methods are satisfactory to achieve good fracture healing [25]. Nonetheless, open reduction has certain advantages in terms of early return of function, better appearance, shorter immobilization and increased patient satisfaction, especially when there is heavy displacement or when there is a complicated fracture pattern [26]. Closed reduction is still an acceptable option in certain clinical situations wherein risks from surgery have weighed more than potential advantages or when fracture patterns are amenable to non-operative care [27]. Meta-analysis of high-level evidence in randomized controlled trial suggests that ORIF significantly improves functional outcomes, decreases pain, and restores occlusion and jaw symmetry. These long-term benefits must be weighed against the increased risk of postoperative infection and exposure of the facial nerve to potential injury [28]. Finally, decisions regarding treatment must be made in accordance with judicious clinical evaluation, patient-specific considerations and the experience of the surgeon. On-going refinement of surgical methods, fixation devices and perioperative management will certainly continue to better optimize outcomes and reduce complications in mandibular fracture care in the years to come.

## Conclusion:

Both open and closed reduction methods are successful in attaining stable results in mandibular fracture treatment. Open reduction has benefits of earlier functional recovery, reduced immobilization and better aesthetic outcomes, but closed reduction is still a valuable alternative in specifically chosen cases with minor displacement. Accurate case selection and tailored treatment planning continue to be key factors in maximizing the patient outcomes.
